# Long non-coding RNA MIR4435-2HG/microRNA-125a-5p axis is involved in myocardial ischemic injuries

**DOI:** 10.1080/21655979.2022.2051259

**Published:** 2022-04-27

**Authors:** Xiuling Wang, Lina Ren, Shuai Chen, Yanli Tao, Dandan Zhao, Chunwei Wu

**Affiliations:** Department of Cardiology, The First Affiliated Hospital of China Medical University, Shenyang110001, Liaoning Province, China

**Keywords:** MI, MIR4435-2HG, miR-125a-5p, MTFP1, apoptosis

## Abstract

This study aimed to investigate whether and how long non-coding RNA (lncRNA) MIR4435-2 host gene (MIR4435-2HG) involved in acute myocardial ischemia/reperfusion (I/R). Blood samples were collected from acute myocardial infarction (AMI) patients to detect MIR4435-2HG expression. *In vivo* myocardial I/R mice model and *in vitro* H_2_O_2_-induced oxidative stress model were established. Echocardiography, TUNEL assay and lactate dehydrogenase (LDH) detection were performed to assess heart infarction and myocardium apoptosis. Relationship among microRNA-125a-5p (miR-125a-5p), MIR4435-2HG and Mitochondrial fission protein 1 (MTFP1) was predicted by Targetscan and verified by luciferase reporter assay. MIR4435-2HG was notably upregulated in AMI patients, myocardial I/R mice and H_2_O_2_-treated cells. Knockdown of MIR4435-2HG notably alleviated infraction volume, ejection fraction (EF) and fractional shortening (FS) levels, cell apoptosis portion and pro-apoptotic cleaved-caspase-3 and Cyt c expression caused by myocardial I/R and oxidative stress, as well as improved cardiomyocytes viability. Transfection with miR-125a-5p alleviated MIR4435-2HG-caused cardiomyocytes apoptosis during oxidative stress. MiR-125a-5p overexpression decreased luciferase activity of the wild-type MIR4435-2HG compared with the mutated MIR4435-2HG. The expression levels of MTFP1 were elevated in myocardium from MI mice model and H_2_O_2_-treated AC16 cardiomyocytes. In addition, miR-125a-5p overexpression inhibited MTFP1 expression, and could stimulate the wild-type MTFP1 promoter luciferase activity but not the mutated one. Our findings revealed the role of MIR4435-2HG in MI-induced myocardium injury and cardiomyocytes apoptosis, disclosed a novel MIR4435-2HG/miR-125a-5p regulatory axis during myocardial I/R, and thus identified a potential target for the therapy of myocardial IR injury.

## Graphical abstract

**Figure:**
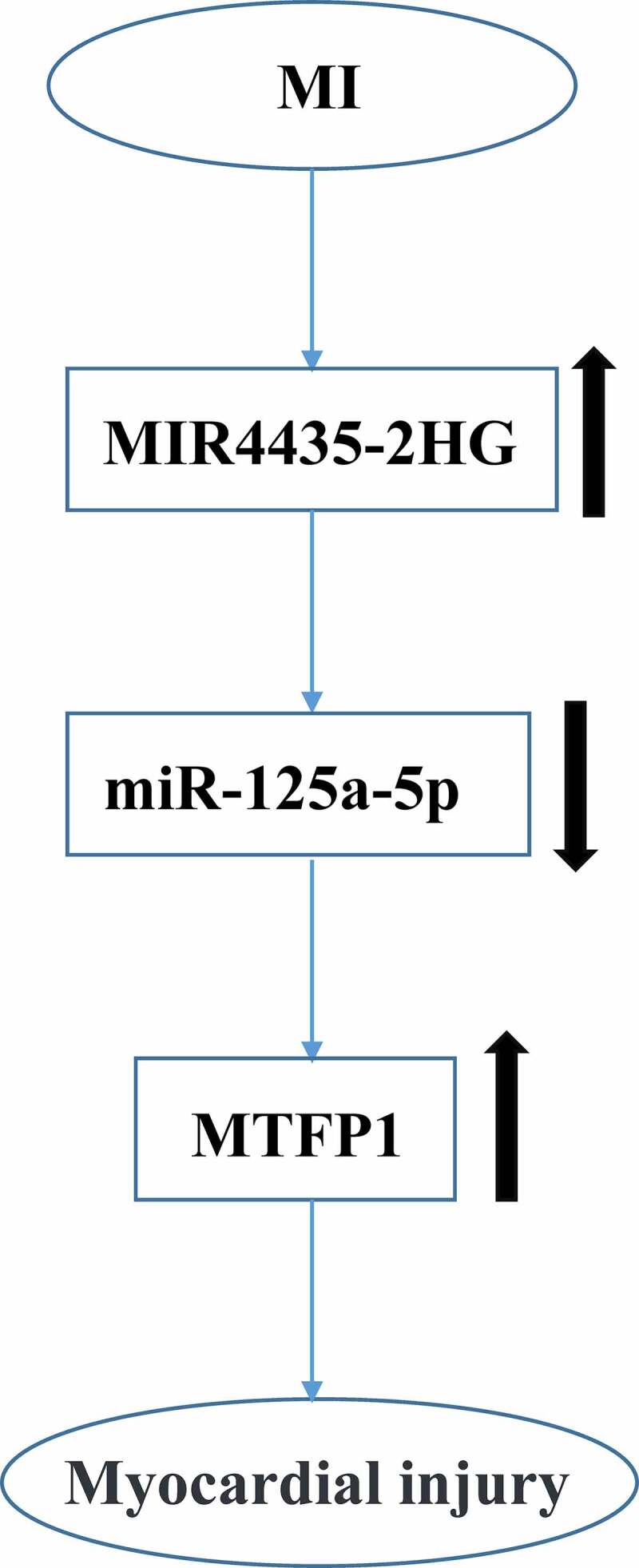

## Highlights

- MIR4435-2HG was upregulated in AMI patients.
- MIR4435-2HG is involved in myocardial injury.
- miR-125a-5p mediated the role of MIR4435-2HG in myocardial injury.
- MIR4435-2HG can regulate the expression of MTFP1.

## Introduction

The incidence of cardiovascular diseases raises with the increasing of age [1]. In a report from World Health Organization, 23 million people may succumb to death caused by cardiovascular diseases by 2030 [1]. Myocardial infarction (MI) is the major cause of cardiovascular diseases [2]. Moreover, reperfusion strategies, such as coronary artery bypass surgery, percutaneous coronary intervention and coronary thrombolysis treatment are the most prevalent treatments for MI [3,4]. However, the myocardial ischemia/reperfusion (IR) following myocardial ischemia therapy may cause severe pathological damages including heart failure, spinal cord and brain function alteration after blood reperfusion, which could reduce the benefit of reperfusion and cause myocardial I/R injury, lead to poor prognosis of post-ischemia [2,5]. MI is known to initiate cell apoptosis cascade and further leads to heart failure [6,7].

MicroRNAs (miRNAs) and non-coding RNAs (lncRNAs) can interact with proteins, DNA and other RNAs, thus play regulatory roles in various cellular physiological processes [8–14]. Increasing evidence showed aberrant lncRNAs and miRNAs expression in cardiovascular disease, metabolic diseases, neurological and cancer progression [15–20]. LncRNA MIR4435-2 host gene (MIR4435-2HG) was found to recruit miRNAs to participate in tumor progression [21,22]. For instance, miRNA-206/YAP1 axis mediates the function of MIR4435-2HG in colorectal cancer cell proliferation via the [23]. MIR4435-2HG participated in prostate cancer and non-small cell cancer progression by regulating TGF-β [24,25]. However, whether and how MIR4435-2HG involved in cardiovascular diseases remain unclear.

Circulating miR-125a-5p was identified as a potential biomarker of endothelial dysfunction and associated with cardiomyopathy genesis [26]. MiR-125a-5p is upregulated at initiation of reperfusion after ischemia and act as cardioprotective factor to facilitate the function of nitric oxide (NO) signaling [27]. Whether miR-125a-5p mediates the regulation of MIR4435-2HG in cardiovascular disease is unknown.

Mitochondrial fission protein 1 (MTFP1) was previously reported to be a pro-apoptotic protein and involved in the regulation of the mTOR signaling [28]. A recent study showed that knockdown of MTFP1 could improve doxorubicin-induced cardiomyopathy [29], suggesting that MTFP1 may be involved in the cardiovascular disease. Moreover, bioinformatics analysis have already revealed that there are binding sites between miRNA-125a-5p and MTFP1. We therefore hypothesized that MTFP1 may be involved in the underlying mechanism of MIR4435-2HG/miR-125a-5p axis in myocardial I/R. Altogether, the study aimed to investigate the role and underlying mechanism of MIR4435-2HG in myocardial I/R.

## Materials and methods

### Patient information

Blood samples were collected from healthy donors (25) and AMI patients (26) admitted at Department of Cardiology, The First Hospital of China Medical University of China Medical University from July 2014 to June 2016. The baseline clinical and biochemical characteristics of individuals are listed in Table 1. Fifty-nine years old is the AMI patients’ mean age. Patients who suffered from chest pain over 12 h, received thrombolytic therapy, or had severe heart failure were excluded from this study. The 25 healthy participants received routine medical examinations at the aforementioned hospital. All individuals in the healthy group showed normal physical data including normal outcomes from electrocardiographic echocardiography and laboratory tests, indicating no signs of cardiovascular disease. All experimental procedures followed the human experiment guideline of Declaration of Helsinki (World Medical Association). All participants signed written consent. Plasma samples were stored at −80°C for future use.

**Table 1.** Baseline clinical and biochemical characteristics of individuals (n = 26)

Variable	N	Value
Age (years)	26	55.9 ± 8.1
Female, %	12	(46.1%)
Diabetes, %	10	(38.5%)
Current or prior smoking, %	15	(57.7%)
Hypertension, %	17	(65.4%)
Hyperlipidemia, %	20	(76.9%)
Prior myocardial infarction, %	3	(11.5%)
Body mass index (kg/m^2^)	26	28.5 ± 6.4
Systolic blood pressure (mmHg)	26	126 ± 21
Diastolic blood pressure (mmHg)	26	73 ± 12
ST-elevation myocardial infarction, %	15	(57.7%)
Aspirin use	21	(80.8%)
Beta blocker use	23	(88.5%)
Angiotensin converting enzyme inhibitor use	16	(61.5%)
Statin use	25	(96.1%)

Values expressed as mean ± standard deviation.

### Animal information

According to the standards of Care and Use enacted by Laboratory Animals of the First Hospital of China Medical Universit, animal experiments were conducted. Male C57BL/6 mice (4–6 months old) were obtained from Vital River Company (Beijing, China), and had free access to water and food and housed at 25°C with 12 h/12 h cycle.

### MI mice model

The myocardial I/R model was built to mimic AMI. For myocardial I/R operation, sodium pentobarbital (30 mg/kg) anesthetized mice, which then were fixed. Electrocardiogram was monitored by Electrocardiographic (ECG). Silk suture (6–0) was used to ligate the left anterior descending coronary artery (LAD). Then, ST-segment elevation and recovered T segment was recorded to judge the successful occlusion. After 30 min blood occlusion, blood supplement was restored for 120 min. Mice got similar operation with no LAD occlusion in the sham group. After myocardial I/R operation, echocardiography measured the cardiac function on the first and third day, then the heart tissues were obtained for other experiments.

### Assessment of area at risk and infarct size

To evaluate the effect of MIR4435-2HG overexpression on the area at risk and infarct size, separate groups of animals were created. After 24 h of coronary artery ligation, 1% Evans Blue dye was perfused into the aorta and coronary arteries, with distribution throughout the ventricular wall proximal to the site of coronary artery ligation. Hearts were frozen for 15 min and cut into four transverse sections below the ligature. These sections were weighed and then incubated with a 1% 2, 3, 5-triphenyltetrazolium chloride (1%, TTC, Sigma, USA) solution at 37°C for 20 min. The infarct area, and the left ventricular (LV) area from each section were analyzed by use of Nikon COOLPIX A900 digital camera, multiplied by the weight of the section, and then totaled from all sections. The ratio of infarct size to area at risk.

### Mice treatment

To evaluate the function of MIR4435-2HG in mice myocardial I/R model, mice were divided into four groups including sham, myocardial I/R model, myocardial I/R + sh negative control (NC), myocardial I/R + shMIR4435-2HG groups. Lentiviral vectors carrying MIR4435-2HG shRNA was designed and got from Shanghai Sangon Biotech Co., Ltd. (Shanghai, China). shMIR4435-2HG sequence is 5'-CTGGAAACCTCTTGACTCT-3'. The shMIR4435-2HG or shNC was injected at a dosage of 1 × 10^7^ TU per mice via inserting a 30-gauge syringe to the left ventricular cavity before ischemia occlusion.

### Echocardiographic measurements

The two-dimensional transthoracic echocardiography was administrated after myocardial I/R surgery. Using the Vevo 707B system of Visual Sonics Company (Toronto, Ontario, Canada), ultrasound was performed after mice were anesthetized. The left ventricular fractional shortening (FS%) and ejection fraction (EF%) were calculated from M-mode equipped by a 30-MHz frequency.

### TUNEL assay

After 24 h of myocardial I/R operation, the heart tissues of mice were collected and fixed in 10% formalin were routinely processed and paraffin embedded. Heart sections (5 μm) were stained with hematoxylin and eosin (H&E) [30]. Then, paraformaldehyde (4%) and paraffin fixed and embedded the slices, using the One Step TUNEL Apoptosis Assay Kit (C1086, Beyotime, Shanghai, China), TUNEL assay was performed following manufacturer’s instructions. As IHC staining, slides were captured and calculated.

### Cell culture

From American Type Culture Collection, human adult ventricular cardiomyocytes AC16 cell line was obtained. DMEM (Gibco, Thermo, MA, USA) with fetal bovine serum (10%, FBS, Gibco), streptomycin (100 μg/mL) and penicillin (100 U/mL) cultured cells in an incubator (37°C) with 5% CO_2_.

### Hypoxia/reoxygenation (H/R) in AC16 cells

To mimic rat myocardial I/R-induced cell apoptosis, an oxidative stress model was constructed. After achieved 80–90% confluence of AC16 cells, which were seeded in wells (1 × 10^5^ cells per well). Then, cells were incubated by DMEM medium with H_2_O_2_ (200 μM) for 4 h.

### Transfection of miRNA and shRNA

The sequence of MIR4435-2HG was designed using the Pubmed website. The shMIR4435-2HG, pcMIR4435-2HG, miR-125a-5p mimic and inhibitor and negative control (NC), were purchased from Ribobio. Sequences were as follows: miR-125-5p mimic, 5’-UCCCUGAGACCCUUUAAUUCCUGUGA-3’; miR-125-5p inhibitor, 5’-UCACAGGUUAAAGGGUCUCAGGGA-3’; NC, anti-sense, 5’-ACAAAGUUCUGUGAUGCACUGA-3’; sense, 5’-ACAAAGUUCUGUGAUGCACUGA-3’; shMIR4435-2HG, 5’-CTGGAAACCTCTTGACTCT-3’; shMTFP, 5’-TTAGGAACCAGGGAGCGGAAA-3’. Cardiomyocytes were seeded in wells (1 × 10^5^ cells) and achieved monoplayer confluence. After 12 h starvation, using Lipofectamine 2000, shMIR4435-2HG, or pcMIR4435-2HG, miR-125a-5p mimic and inhibitor (50 nmol/L) were transfected into cells. Then, cells were harvested for the subsequent experiments after transfection of 48 h. Lentivirus (carrying shRNA for lncMIR4435-2HG) was injected into the caudal vein 14 d before I/R operation.

### LDH activity detection

Lactate dehydrogenase (LDH) is regarded as one of biomarkers related to myocardial I/R. Following the manufacturer’s instructions, we detected LDH levels using the LDH Release Assay Kit (Beyotime, C0017, Shanghai, China).

### MTT assay

Cells (1 × 10^3^) were plated and treated with H_2_O_2_ alone, or together with shMIR4435-2HG, miR-125a-5p and pcMIR4435-2HG as indicated. MTT (20 μL, 5 mg/mL) was added at the corresponding time point. The culture medium was discarded after a 4-h reaction, and DMSO (150 μL) was added for 15 min in a shaker. Then, the absorbance values at 450 nm was detected.

### RNA extraction and real-time PCR

This experiment was performed as described [31]. After TRIzol Reagent extracted the total RNAs, cDNA synthesis Kit was used to reverse the RNAs to cDNAs. Then, SYBR Green Super Mix kit performed the gene expression detection with GAPDH and U6 as the internal control, respectively, and determined using the 2^−ΔΔCt^ method. The primers were: U6, 5'-GCTTCGGCAGCACATATACTAA-3', 5'-AACGCTTCACGAATTTGCGT-3' ; miR-125a-5p, 5' - ACACTCCAGCTGGGTCCCTGAGACCCTTTAAC-3' , 5' - TGTCGTGGAGTCGGCAATTC-3' ; GAPDH: 5' -AAGAAGGTGGTGAAGCAGGC-3' and: 5' -TCCACCACCCAGTTGCTGTA-3' ; MTFP1: 5' - TAATCCACCCCATCGACAG-3' and: 5' - TCCACTGACGGGTACAGCTT-3' ; MIR4435-2HG, 5' -AGAATGAAGGCTG AGGTGTG-3' , 5' -CAGCGACCATCCAGTCATTTA-3' .

### Western blotting

After myocardial I/R operation, myocardium tissues were lysed by RIPA lysis buffer. After BCA kit determined the protein concentration, 35 ug protein samples were separated by SDS-polyacrylamide gel (10%, PAGE) and transferred to PVDF membrane, which was blocked with 5% nonfat milk for 1 h at room temperature. Then, primary antibodies (anti-cleaved-caspase-3; anti-caspase-3; anti-Cyt c; anti-MTFP1; anti-GAPDH) incubated the membrane at 1:1000 dilution from Abcam overnight at 4°C. The corresponding secondary antibodies conducted for 2 h at room temperature. ECL kit was used to visualize the signals and images were captured.

### RNA pull-down assay

RNA pull-down assay was performed as described [32]. Biotin-labeled miR-125a-5p (Bio-miR-125a-5p), biotin-labeled mutated miR-125a-5p (Bio-miR-125a-5p Mut) and biotin-labeled negative control (Bio-NC) were obtained from Ribobio. DMEM medium with FBS (10%) cultured Hela cells, which were transfected with Bio-NC, Bio-miR-125a-5p or Mut for 48 h. After cells were lysed, streptavidin Magnetic Beads were added at 4°C for 2 h. The coprecipitated RNA were separated from biotinylated nucleic acids coated beads, and used for qRT-PCR analysis.

### Target prediction

The binding sites between miRNA-125a-5p and MTFP1 was predicted by bioinformatics analysis tool Targetscan (http://www.targetscan.org).

### Luciferase reporter gene assay

Luciferase reporter gene assay was performed as described [33]. MIR4435-2HG and MTFP1 mRNA sequence were designed on Pubmed website. 293 T cell line was cultured in DMEM with FBS (10%). After pGL3 vector carried wild-type and mutated sequence of MIR4435-2HG or wild-type and mutated sequence of MTFP1, respectively, Lipofectamine 3000 was used to transfected MIR4435-2HG WT, MIR4435-2HG MUT, MTFP1-WT or MTFP1-MUT and miR-125a-5p mimics or NC to 293 T cells, and internal control was PRL-TK plasmid. Finally, using a dual luciferase reporter assay kit, the luciferase activities were measured at 48 h post-transfection.

### Statistical analysis

The data were analyzed with Graphpad prism 7 software and were presented as the mean + standard deviation (SD). *p* < 0.05 was considered as statistical significance. The comparison between two groups was performed by using Student’s t-test, while difference among multiple groups was analyzed by using one-way ANOVA. Post hoc test was performed by Tukey’s method.

## Results

In this study, we aimed to investigate whether and how MIR4435-2HG involves in myocardial I/R, and our finding suggested a potential MIR4435-2HG/miR-125a-5p axis to accelerate myocardial injury during myocardial I/R injury via promoting apoptosis

### LncMIR4435-2HG was upregulated in myocardial I/R

**Figure 1.**
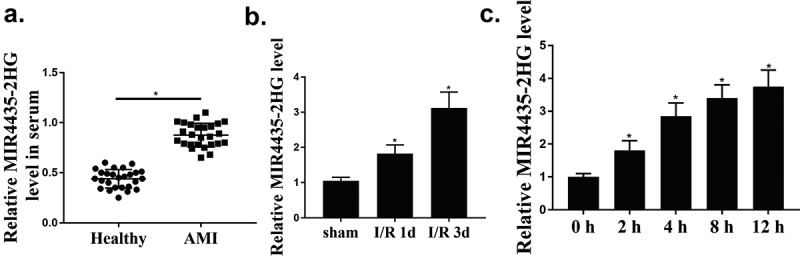
The expression levels of MIR4435-2HG during myocardial I/R-induced injury. (a) qRT-PCR detection of MIR4435-2HG in AMI patient (n = 26) and healthy donors (n = 25) blood samples. (b) Elevated expression levels of MIR4435-2HG in heart tissues of mice were detected by qRT-PCR at 1 and 3 days after myocardial I/R (n = 6). (c) MIR4435-2HG expression in AC16 cells after H_2_O_2_ treatment for 0, 2, 4, 8, 12 h. *P < 0.05 vs. Healthy, sham or PBS, Three independent experiments were repeated. n = 3.

Firstly, we assessed the role of MIR4435-2HG in myocardial I/R. The results showed that MIR4435-2HG expression were elevated in AMI patients compared with that in healthy donors (*p* < 0.05) (Figure 1a). Then myocardial I/R mice model and a cellular oxidative stress model were established. MIR4435-2HG expression in peri-infarction were remarkably elevated on the first and third day after myocardial I/R (Figure 1b, *p* < 0.05). Consistently, H_2_O_2_-induced oxidative stress increased MIR4435-2HG expression in a time-dependent manner in AC16 cells (Figure 1c, *p* < 0.05). Altogether, these suggest that MIR4435-2HG is overexpressed in myocardial I/R.

### Knockdown of lncMIR4435-2HG protected myocardium from injury

**Figure 2.**
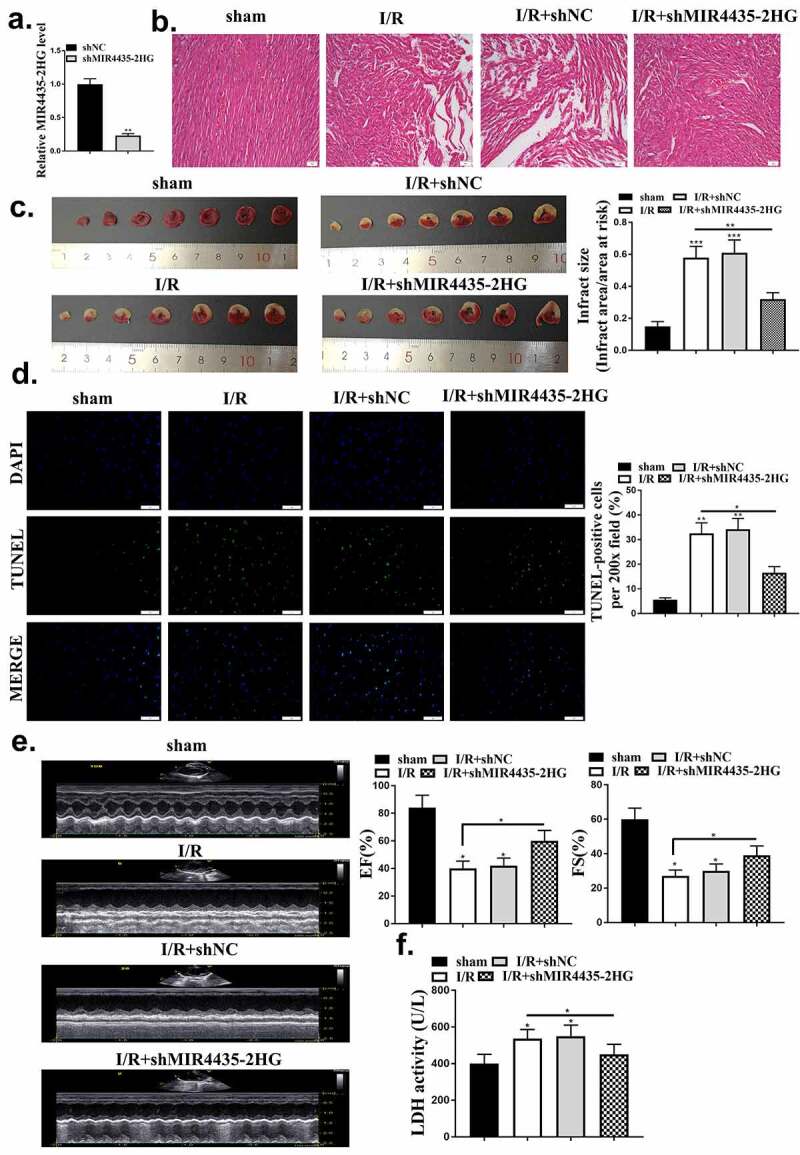
Knockdown of MIR4435-2HG approves myocardial I/R-induced injury. (a) MIR4435-2HG expression detected by qRT-PCR after injection of shMIR4435-2HG lentiviral vectors or shNC in mice left ventricular chamber. (b) HE staining of mice heart tissues in myocardial I/R model. sham, the control group; myocardial I/R, mice myocardial ischemia treatment; myocardial I/R + shNC, myocardial I/R model with injection of negative control vector; myocardial I/R + shMIR4435-2HG, myocardial I/R model with injection of shMIR4435-2HG. (c) Hearts were sliced and stained with TTC. (d) TUNEL assay, and quantification shown in diagram. (e) Echocardiography examination of EF (ejection fractions) and FS (fractional shortening) after myocardial I/R and knockdown of MIR4435-2HG. (f) Serum level of LDH in myocardium. *P < 0.05, **P < 0.01 vs. sham or as indicated. Three independent experiments were repeated. n = 5 mice.

To further evaluate the function of MIR4435-2HG in myocardial I/R mice, knockdown of MIR4435-2HG was performed by injection of shMIR4435-2HG lentiviral vectors into the myocardial I/R model. The efficacy of shMIR4435-2HG was confirmed by decreased expression levels of MIR4435-2HG in mice heart tissues (*p* < 0.01) (Figure 2a). HE staining results showed an obvious myocardium injury after myocardial I/R, and there was a significant decline with the knockdown of MIR4435-2HG (Figure 2b). After reperfusion, heart slices were stained with TTC to examine myocardium infarct size, and it showed that knockdown of MIR4435-2HG significantly reduced the infarct size (*p* < 0.01) (Figure 2c). Moreover, it was found that myocardial I/R increased cell apoptosis rate, which could be effectively reduced (*p* < 0.05) by knockdown of MIR4435-2HG (Figure 2d). As shown in Figure 2e, echocardiography examination showed that the percentage of EF and FS were obviously decreased during myocardial I/R treatment (*p* < 0.05), and knockdown of MIR4435-2HG abolished this effect (*p* < 0.05). We also detected the secretion level of LDH, the biomarker of cardiomyocyte injury. Similarly, knockdown of MIR4435-2HG could remarkably block myocardial I/R-induced LDH elevation (*p* < 0.05) (Figure 2f).

### The cardio-protective role of downregulation of MIR4435-2HG in oxidative stress-induced apoptosis in vitro

**Figure 3.**
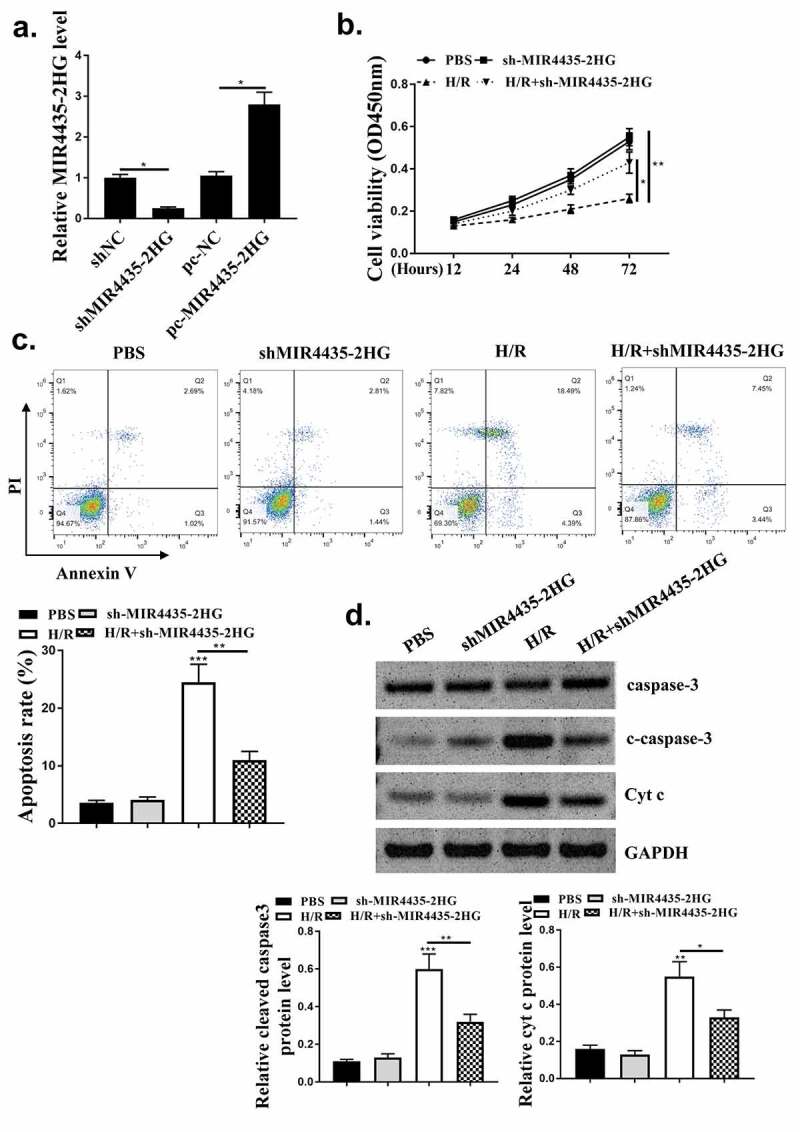
Inhibition of MIR4435-2HG attenuates apoptosis in oxidative stress cell model. (a) qRT-PCR determined the efficiency of shMIR4435-2HG and pc-MIR4435-2HG. (b) AC16 cell viability detected by MTT assay. (c) AC16 cell apoptosis was detected by Flow cytometry. (e) Western blotting analysis to determine cleaved caspase-3 (c-caspase-3), caspase-3 and cytochrome c (Cyt c) expression. *P < 0.05, **P < 0.01. Three independent experiments were repeated, and the representative results were presented. n = 3.

After evaluating the role of MIR4435-2HG in mice myocardial I/R model, we further explored its pro-apoptotic function in AC16 cardiomyocytes. The efficiency of MIR4435-2HG knockdown and overexpression was tested (Figure 3a, *p* < 0.05). MTT assay results showed H_2_O_2_ treatment obviously impaired cell viability (*p* < 0.01), and this impairment could be reversed by knockdown of MIR4435-2HG (*p* < 0.05) (Figure 3b). Cell apoptosis induced by H_2_O_2_ treatment (Figure 3c, *p* < 0.01) could also be blocked by knockdown of MIR4435-2HG (*p* < 0.05). Furthermore, pro-apoptotic proteins, cleaved-caspase 3 and Cyt c expression were also elevated (*p* < 0.01) in H_2_O_2_ oxidative stress model and decreased (*p* < 0.05) by MIR4435-2HG knockdown (Figure 3d).

### MIR4435-2HG sponged miR‑125a-5p

**Figure 4.**
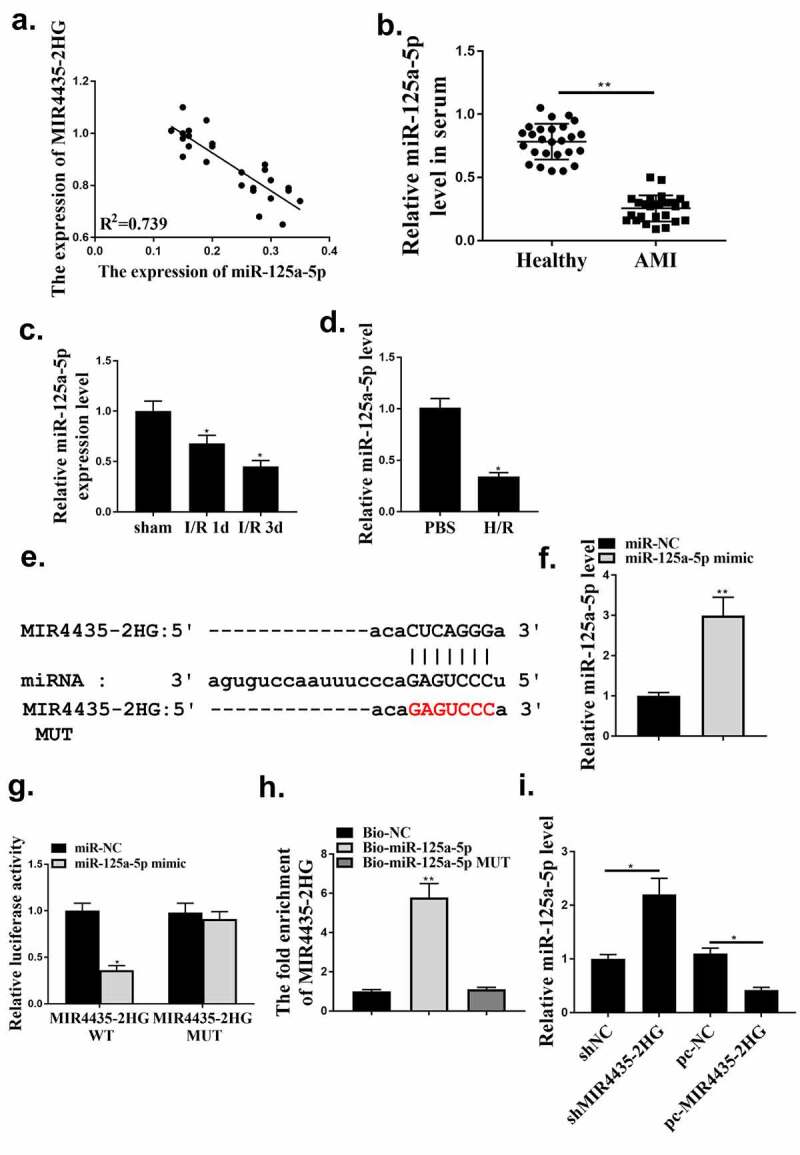
LncRNA MIR4435-2HG functions as miR-125a-5p sponge in cardiomyocytes. (a) The correlation between MIR4435-2HG and miR-125a-5p expression was analyzed in AMI patient blood samples. n = 26. (b) The expression of miR 125a-5p in the serum of AMI patients. (c) qRT-PCR experiment to evaluate miR-125a-5p expression in mice myocardial I/R model at the first and third day after myocardial I/R. (d) qRT-PCR experiment to evaluate miR-125a-5p expression in H_2_O_2_-induced oxidative stress model of AC16 cells. (e) Binding site prediction between MIR4435-2HG mRNA and miR-125a-5p. (f) qRT-PCR experiment to evaluate miR-125a-5p expression after transfection of miR-125a-5p mimic. (g) The luciferase activity was detected. (h) RNA pulldown assay. (i) qRT-PCR experiment to evaluate miR-125a-5p expression after transfection of shMIR4435-2HG or pc-MIR4435-2HG. *P < 0.05, **P < 0.01. Three independent experiments were performed. n = 3.

Next, the underlying mechanisms involved in the regulation of MIR4435-2HG in myocardial I/R were explored. The results indicated that MIR4435-2HG and miR-125a-5p expression in serum of the 26 AMI patients showed a negative correlation (R^2^ = 0.739) (Figure 4a). Then miR-125a-5p expression in the serum of AMI patients, myocardial I/R mice model and oxidative stress-induced cell injury model was detected. As shown in Figure 4b, the expression of miR 125a-5p in the serum of AMI patients was reduced. Moreover, miR-125a-5p expression notably reduced on the 1st and 3rd day post-myocardial I/R (Figure 4c, *p* < 0.05). Consistent with the *in vivo* results, miR-125a-5p expression were obviously decreased in H_2_O_2_ treated AC16 cells (Figure 4d). Besides, bioinformatics analysis predicted a putative binding site of miR-125-5p on MIR4435-2HG (Figure 4e). Thus, we next tried to testify the interaction between miR-125-5p and MIR4435-2HG via luciferase gene assay and RNA pulldown. The efficacy of miR-125-5p mimics was confirmed (p < 0.01) (Figure 4f). Results showed that miR-125a-5p mimics treatment could reduce the luciferase activity of the wild type MIR4435-2HG, but not the mutated MIR4435-2HG reporter plasmid (*p* < 0.05) (Figure 4g). In addition, in Figure 4h, Bio-miR-125a-5p transfection could increase MIR4435-2HG expression, while mutated miR-125a-5p had no effect. Furthermore, MIR4435-2HG knockdown or overexpression could upregulate or downregulate miR-125a-5p, respectively (Figure 4i). These indicate that MIR4435-2HG functioned as miR-125a-5p sponge.

### MIR4435-2HG regulated AC16 cell viability and apoptosis after H_2_O_2_-induced injury through miR-125a-5p

**Figure 5.**
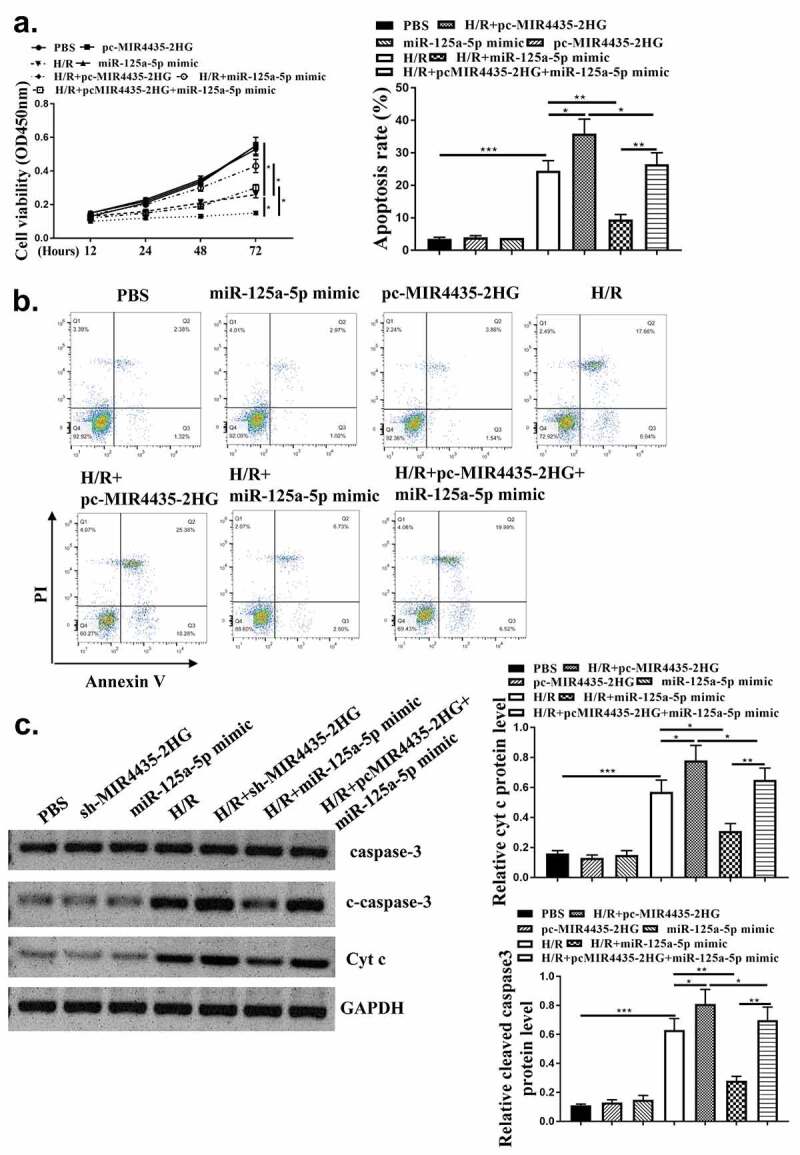
MiR-125a-5p mediates MIR4435-2HG function. (a) MTT assay of AC16 cells. (b) Flow cytometry of AC16 cell. (c) caspase-3 and cytochrome c (Cyt c) were detected by western blotting. Three independent experiments were performed. n = 3. *P < 0.05.

To explore whether miR-125a-5p mediated MIR4435-2HG functions in myocardial I/R, pc-MIR4435-2HG and miR-125a-5p alone or in combination were transfected into AC16 cells, followed by H_2_O_2_ treatment. Then cell viability and apoptosis were detected. As shown in Figure 5a, transfection with pc-MIR4435-2HG induced enhanced inhibition of cell viability under H_2_O_2_ treatment (*p* < 0.05), which was reversed by co-transfection with miR-125a-5p mimics (*p* < 0.05). Flow cytometry assay (Figure 5b) and Western blot analysis on apoptosis-related biomarkers cleaved caspase-3 and Cyt c (Figure 5c) (*p* < 0.05) showed elevated apoptosis rate with MIR4435-2HG overexpression and decreased apoptosis rate with miR-125a-5p mimics. These together indicate that miR-125a-5p functions as a mediator of MIR4435-2HG during H_2_O_2_-induced injury of cardiomyocytes.

### MiR‐125a-5p targeted MTFP1 in cardiomyocytes

**Figure 6.**
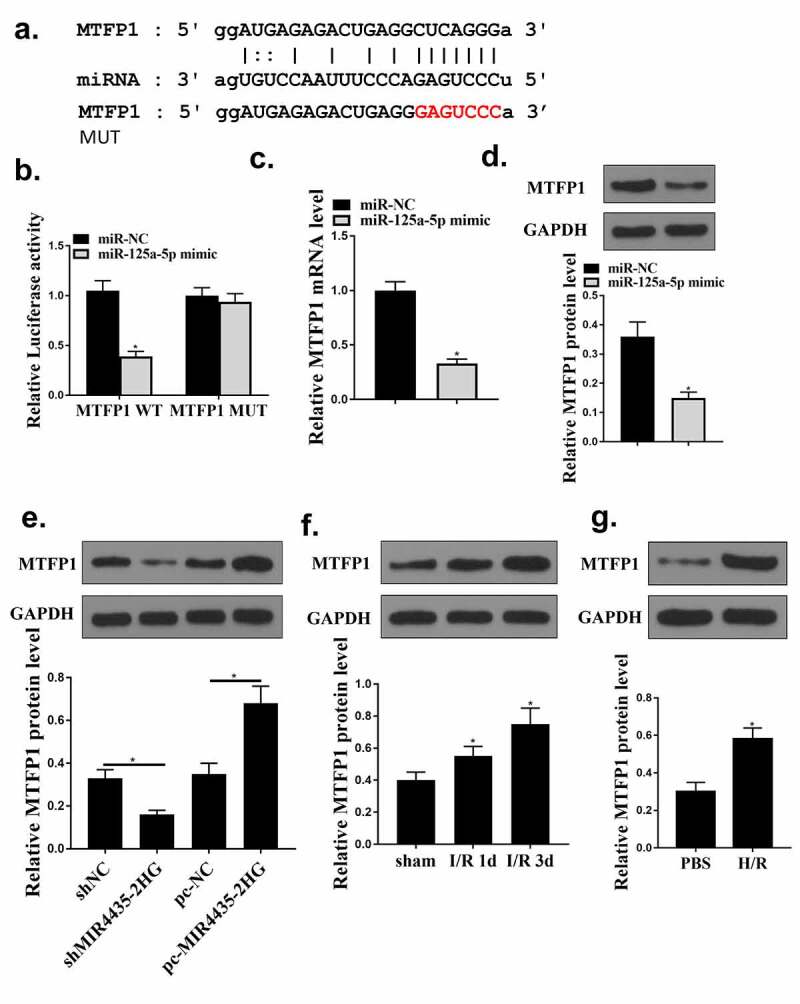
MiR‐125a-5p targets MTFP1. (a) Bioinformatics prediction. (b) Luciferase activity was evaluated and calculated in diagrams. (c-d) qRT-PCR and Western blot analysis to detect MTFP1 mRNA (c) and protein (d) levels in AC16 cells. (e) Western blotting experiment detect the MTFP1 level in AC16 cells. (f) The expression of MTFP1 in myocardium. (g) MTFP1 expression in AC16 cells. *P < 0.05 vs. NC, sham or as indicated. Three independent experiments were repeated. n = 3. The relative protein levels were shown in histograms.

To identify the mechanism of miR-125a-5p in myocardial I/R injury, bioinformatics analysis was used and it predicted a putative binding site between MFTP1 and miR-125a-5p (Figure 6a). Co-transfection with miR-125a-5p mimics significantly decreased MTFP1 WT activity (*p* < 0.05) but not MTFP1 MUT activity, indicating there was an interaction between miR-125a-5p and MTFP1 (Figure 6b). Moreover, miR-125a-5p mimics significantly reduced MTFP1 expression (Figure 6c,d, *p* < 0.05) in cardiomyocytes. Besides, MTFP1 expression were decreased with knockdown of MIR4435-2HG, while elevated with the overexpression of MIR4435-2HG (Figure 6e) (*p* < 0.05). Next, the expression of MTFP1 in myocardial I/R mice model and H_2_O_2_ cell model was detected. MTFP1 expression were increased in mice model (*p* < 0.05) (Figure 6f) and cells with H_2_O_2_ treatment (Figure 6g) (*p* < 0.05). These suggest that MTFP1 is involved in the role of MIR4435-2HG/miR-125a-5p axis in myocardial I/R injury.

## Discussion

Cardiovascular diseases especially myocardial I/R causes high morbidity and mortality globally. And despite the developing therapeutic strategies, current therapies such as surgery therapy, often leads to myocardial I/R injury and severe after effects [4]. The mechanisms of myocardial I/R injury were generally described, upon ischemia, blood supplement for heart was blocked, which caused reduced oxygen supply, increased mechanical stress like excess reactive oxygen species (ROS) and ATP, as well as overload of unfold and misfold proteins [34,35]. These internal stress further leads to hyperactivation of calcium uptake and unfolded protein responses, which causes severe endoplasmic reticulum and mitochondria dysfunction, finally cardiomyocyte undergoes uncontrolled death and causes heart failure [36–38].

During multiple pathogenesis of cardiovascular diseases including myocardial I/R injury, cells death was a critical factor causing death [39]. The detection of anti-apoptotic factors like Bcl-2 family members Bcl-xl and Bcl-2 implied an initiation of apoptosis during myocardial I/R, followed by notable rise during reperfusion process [6,7]. These studies revealed accelerated apoptosis of myocardium cell during reperfusion following myocardial I/R. Furthermore, it was verified that the administration of broad-spectrum inhibitor targeting pro-apoptotic factors, including caspase-2, caspase-3 and caspase-7, at initial time of reperfusion could lead to shrunken size of post-ischemia infarct [40]. Nevertheless, previous studies indicated that single mitochondrial-targeting drug has limited benefits for clinical outcomes. Hence, it becomes urgent to explore novel regulatory mechanisms and adjuvant therapy manners for myocardial I/R injury.

Extensive studies have demonstrated the role of ncRNAs during myocardial I/R injury [41]. Besides, lncRNAs and miRNAs are more stable and detectable in body fluids, make them promising candidates as diagnostic signatures in diseases. Numerous studies have highlighted miRNAs can be functioned as biomarkers for cardiovascular disease diagnosis, however, less is known about the diagnostic and prognostic roles of lncRNAs. Though, emerging evidence has proved the critical roles of lncRNAs in the development of cardiovascular diseases [42,43]. LncRNA MIR4435-2HG is widely studied in cancers. For instance, MIR4435-2HG participated in progression of colorectal cancer, prostate cancer and non-small cell cancer, regulated the function of TGF-β [23–25]. However, the role of MIR4435-2HG in cardiovascular disease has not been investigated. Our study detected a notably higher expression levels of MIR4435-2HG in AMI patient blood samples compared with healthy donors, and subsequent myocardial I/R mice model as well as H_2_O_2_-induced cardiomyocytes apoptosis confirmed the connection between the upregulation of MIR4435-2HG and myocardial I/R. The surveillance of heart function by echocardiography and detection of apoptotic cells indicated that knockdown of MIR4435-2HG could effectively reverse myocardial I/R-caused heart infarction and cardiomyocytes injury, presented by recovered EF and FS, decreased portion of apoptotic cell and level of cleaved caspase-3, pro-apoptotic proteins and Cyt c. Considering the major regulatory role of lncRNAs in interacting with miRNAs, we next investigated if MIR4435-2HG functioned through miRNA.

MiR-125a-5p has been reported to be involved in traumatic osteoarthritis [44] and also known to be related with heart function. For example, a bioinformatics analysis indicated the connection between miR-125a-5p and cardiomyopathy genesis, promoted circulating miR-125a-5p in plasma [26]. MiR-125a-5p was upregulated at reperfusion initiation after ischemia and acted as a cardioprotective factor to facilitate the function of nitric oxide (NO) signaling [27]. Our findings showed a reduced miR-125a-5p expression in injured heart tissues after ischemia and reperfusion, and transfection with miR-125a-5p mimics could improve cardiomyocyte survival and impair H_2_O_2_-caused apoptosis. Meanwhile, there is a negative correlation between MIR4435-2HG and miR-125a-5p. Luciferase assay and RNA pulldown assay further confirmed MIR4435-2HG was a miR-125a-5p sponge. Besides, miR-125a-5p mimics reversed MIR4435-2HG-caused cardiomyocytes damage. These findings suggest that miR-125a-5p may mediate MIR4435-2HG function during myocardial I/R injury.

MTFP1 is a potential pro-apoptotic protein, and a recent study demonstrated that knockdown of MTFP1 could improve doxorubicin-induced cardiomyopathy [28,29]. Here, we predicted a potential binding site of miR-125a-5p on the 3’-UTR of MTFP1 and observed a decreased MTFP1 expression upon transfection with miR-125a-5p. Moreover, an alteration in the expression levels of MTFP1 was found to be followed the knockdown and overexpression of MIR4435-2HG. These results suggest that the roles of MIR4435-2HG and miR-125a-5p in myocardial I/R injury may be mediated by MTFP1. However, whether and how MTFP1 mediated the downstream signaling pathway of MIR4435-2HG/miR-125a-5p axis during myocardial I/R injury needs to be further investigated.

## Conclusion

Our finding suggested a potential MIR4435-2HG/miR-125a-5p axis to accelerate myocardial injury during myocardial I/R injury via promoting apoptosis (graphical abstract), therefore presented evidence for diagnosis and prognosis prediction of myocardial I/R injury therapy.

## Data Availability

The analyzed data sets generated during the study are available from the corresponding author on reasonable request.
